# An Epistatic Interaction between *Pnpla2* and *Lipe* Reveals New Pathways of Adipose Tissue Lipolysis

**DOI:** 10.3390/cells8050395

**Published:** 2019-04-29

**Authors:** Xiao Zhang, Cong Cong Zhang, Hao Yang, Krishnakant G. Soni, Shu Pei Wang, Grant A. Mitchell, Jiang Wei Wu

**Affiliations:** 1 Key Laboratory of Animal Genetics, Breeding and Reproduction of Shaanxi Province, College of Animal Science and Technology, Northwest A&F University, Yangling 712100, China; zhangxiaoo509@163.com (X.Z.); zhangcongcong@nwafu.edu.cn (C.C.Z.); 2 Division of Medical Genetics, Department of Pediatrics, Université de Montréal and CHU Sainte-Justine, 3175 Côte Sainte-Catherine, Montreal, QC H3T 1C5, Canada; yhao8712@gmail.com (H.Y.); shupei.wang@recherche-ste-justine.qc.ca (S.P.W.); 3 Section of Gastroenterology, Hepatology and Nutrition, Department of Pediatrics, Baylor College of Medicine and Texas Children’s Hospital, Houston, TX 77030, USA; krishnakantsoni@yahoo.com

**Keywords:** transacylation, triglycerides/diacylglycerol, enzymology/enzyme mechanisms, lipolysis and fatty acid metabolism, lipids, obesity

## Abstract

White adipose tissue (WAT) lipolysis contributes to energy balance during fasting. Lipolysis can proceed by the sequential hydrolysis of triglycerides (TGs) by adipose triglyceride lipase (ATGL), then of diacylglycerols (DGs) by hormone-sensitive lipase (HSL). We showed that the combined genetic deficiency of ATGL and HSL in mouse adipose tissue produces a striking different phenotype from that of isolated ATGL deficiency, inconsistent with the linear model of lipolysis. We hypothesized that the mechanism might be functional redundancy between ATGL and HSL. To test this, the TG hydrolase activity of HSL was measured in WAT. HSL showed TG hydrolase activity. Then, to test ATGL for activity towards DGs, radiolabeled DGs were incubated with HSL-deficient lipid droplet fractions. The content of TG increased, suggesting DG-to-TG synthesis rather than DG hydrolysis. TG synthesis was abolished by a specific ATGL inhibitor, suggesting that ATGL functions as a transacylase when HSL is deficient, transferring an acyl group from one DG to another, forming a TG plus a monoglyceride (MG) that could be hydrolyzed by monoglyceride lipase. These results reveal a previously unknown physiological redundancy between ATGL and HSL, a mechanism for the epistatic interaction between *Pnpla2* and *Lipe*. It provides an alternative lipolytic pathway, potentially important in patients with deficient lipolysis.

## 1. Introduction

Adipose tissue lipolysis degrades triglycerides (TGs), releasing fatty acids and glycerol. Lipolysis is crucial for energy homeostasis, especially under fasting conditions. Abnormalities of lipolysis have been related to multiple components of metabolic syndrome, including obesity, fatty liver and diabetes [1,2,3,4]. The treatment of inborn errors of beta-oxidation and ketone body metabolism involves the suppression of lipolysis [5,6], but some inborn errors such as deficiencies of pyruvate dehydrogenase or of GLUT1 and other forms of epilepsy, are treated in part by stimulation of lipolysis [7,8,9,10,11]. Lipolysis has been extensively studied, especially in white adipose tissue (WAT) [2,3,12,13,14]. A view has emerged of lipolysis as a linear pathway catalyzed by three enzymes: adipose triglyceride lipase (ATGL) [15], hormone-sensitive lipase (HSL) [16] and monoglyceride lipase (MGL) [17]. ATGL preferentially cleaves TG to diacylglycerols (DGs) [15,18]. HSL has highest lipase activity towards DGs [19], producing monoglycerides (MG). Finally, MGL cleaves MGs to fatty acid (FA) and glycerol [20]. 

Consistent with this model, ATGL-deficient mice accumulate high levels of TG in the key organs of FA flux: adipose tissues, liver and muscle [18]. Also, HSL-deficient mice show high levels of DG accumulation in adipose tissue [19]. We produced mice with combined deficiencies of ATGL and HSL in adipose tissue (DAKO mice), expecting a similar phenotype as in mice with isolated adipose ATGL deficiency (ATGLAKO mice). Surprisingly, the phenotype of DAKO mice differs markedly from that of ATGLAKO mice: 100% of DAKO mice develop liposarcomas, whereas this does not occur in ATGLAKO mice [3]. Furthermore, DAKO mice are more sensitive than ATGLAKO mice to cold stimulation and fasting stress. Together, these observations are incompatible with the notion of a single linear lipolytic pathway [21]. One possible mechanism would be a physiological interaction between ATGL and HSL, such that each enzyme could compensate for the absence of the other. To test this hypothesis, radiolabeled TG and DG substrates were used to test whether (1) in the absence of ATGL, HSL might hydrolyze TG, partially compensating for the TG hydrolase activity of ATGL; and (2) whether in the absence of HSL, ATGL might act on DG, partially compensating for the DG hydrolase activity of HSL. Our results reveal a new branched lipolytic pathway, which may have general importance for energy homeostasis.

## 2. Materials and Methods

### 2.1. Animals and Treatment

Five mouse strains were used: C57BL/6J mice (wild type mice, WT), HSL flox/flox mice, mice deficient in adipose tissue for ATGL (ATGLAKO), HSL (HSLAKO) or both (DAKO). ATGLAKO mice were homozygous for a floxed allele of the *Pnpla2* gene and harbored a transgene, from which Cre is expressed from a *Fabp4* promoter, which is active in adipose tissues. Further details about the creation of ATGLAKO mice were described in our previous publication [22]. HSLAKO mice were homozygous for a floxed allele of *Lipe* and harbored a transgene, from which Cre is expressed from a *Fabp4* promoter. Further details about the creation of HSLAKO mice were described in our previous publications [3]. DAKO mice were created by breeding of the two single knockout lines as we previously described [3]. Each targeted allele had been transferred for at least 8 generations to a C57BL/6J background. 

Mice were raised in a light–dark cycle, with lights on at 07:00 and off at 19:00, and free access to water and food (Teklad Global Rodent, 18% protein; Harlan Laboratories, Madison, WI, USA). Mice were housed at 21 °C unless otherwise specified. Before sacrifice, 3-month-old male mice were fasted for either 5 h or 24 h, anesthetized with pentobarbital sodium (Somnotol; MTC Pharmaceuticals, Hamilton, Ontario, Canada), then WAT was rapidly removed and frozen.

All efforts were made to minimize animal suffering and to reduce the number of animals used in the experiments. Experiments were approved by the Animal Facility Committee of CHU Sainte-Justine Hospital (protocol 620) according to the guidelines of the Canadian Council on Animal Care (http://www.ccac.ca/en/).

### 2.2. TG Hydrolase Activity Assay

About 0.3 g of perigonadal fat from 24-h fasted mice was gently homogenized in 2 mL homogenate buffer (0.5 M sucrose, 1 mM EDTA, 20 mM Tris-HCl pH 7.4, 1 mM DTE, 20 µg/mL leupeptin, 2 µg/mg antipain and 1 µg/mL pepstatin) using a dounce homogenizer. After centrifugation at 1000× *g* for 10 min, aqueous supernatant was removed, then centrifuged at 12,000× *g* for 15 min. The supernatant was taken for the TG hydrolase assay as described [23] with minor modifications. For substrate preparation, 0.05 µg glycerol tri[9,10 (n)-^3^H]oleate (GE Healthcare Bio-Sciences, Baie d’Urfé, Quebec, Canada), 147.5 µg non-labelled triolein (final specific activity, 5.74 µCi/µmol) and 15 µg phosphatidylcholine/ phosphatidylinositol (PC/PI 3:1 *w*/*w*) were mixed. After drying under nitrogen, the mixture was emulsified by sonication in 75 µL 0.1 M potassium phosphate, pH 7.0, and 25 µL 20% defatted BSA in 0.1 M potassium phosphate, pH 7.0. Before the assay, homogenates were pre-incubated in the presence or absence of the ATGL inhibitor Atglistatin (Millipore, Etobicoke, Ontario, Canada, 40 µmol/L) [24], or the HSL inhibitor Bay (50 nM) [25,26], in a shaking water bath at 37 °C for 15 min, then mixed with the substrate. For each assay, 100 µL substrate with 50 µL homogenate and 50 µL reaction buffer (20 mM potassium phosphate, 1 mM EDTA, 1 mM DTE and 0.02% defatted BSA, pH 7.0) were incubated for 30 min at 37 °C. Reactions were terminated by adding 3.25 mL methanol/chloroform/heptane (10/9/7, *v*/*v*/*v*) and 1 mL 0.1 M potassium carbonate, 0.1 M boric acid (pH 10.5), followed by vigorous vortexing. Following centrifugation at 800× *g* for 15 min, scintillation counting was performed on 1 mL of the top heptane phase, which contained radiolabeled fatty acids.

### 2.3. Transacylation Assay

Transacylation was measured as described [27,28,29], by determining the formation of TGs from radioactive DGs. The assay was performed with proteins extracted from the lipid droplet fraction. The isolation of lipid droplets was as described with minor modification [30]. Briefly, 200 mg perigonadal fat was homogenized in protein lysis buffer (0.25 M sucrose, 1 mM EDTA, 10 mM Tris pH 7.0) and 1 mM DTE with protease inhibitors (40 µg/mL leupeptin, 4 µg/mL antipain and 2 µg/mL pepstatin), then centrifuged at 10,000× *g* for 1h. The upper fat layer was carefully taken. Protein extraction from the fat cake was as described [31]. For each assay, 20 µg of the above protein extract was diluted to 100 µL with buffer containing 0.25 mol/L sucrose in 20 mmol/L Tris-HCl pH 7.5, 150 mmol/L NaCl, 0.5 mmol/L EDTA, 0.5% CHAPS and 1 mmol/L DTE. Protein extracts were pre-incubated in the presence or absence of 40 µmol/L ATGL inhibitor (Atglistatin, Millipore, Etobicoke, Ontario M9W 6Y1, Canada), 1µmol/L MGL inhibitor (JZL184, MCE, Monmouth Junction, NJ 08852, USA), or 50 µmol/L DGAT2 inhibitor (JNJ-DGAT2-A, Tocris, Shanghai, China) [32,33,34], in a shaking water bath at 37 °C for 15 min before mixing with substrate. The substrate was prepared as follows: 50 nmol 1,3-dioleoyl-sn-glycerol (Sigma, St. Louis, MO, USA) including 1 µCi (0.05 nmol) [9.10-3H] 1,3-dioleoyl-sn-glycerol (American Radiolabeled Chemicals, St. Louis, MO, USA), 11.25 µg phosphatidylcholine (dissolved in ethanol) and 1.34 mg Na-taurocholate (dissolved in ethanol) were dried under nitrogen. A volume of 1.0 mL potassium phosphate buffer (0.1 mol/L, pH 7.0) was added and sonicated twice for 1 min (Sonic Dismembrator, Fisher Scientific, Ottawa, Ontario K2E 7L6, Canada, power setting 60), with the two sonications separated by 1 min on ice. A volume of 100 µL substrate and a 100-µL sample were mixed and incubated at 37 °C for 10 min. The reaction was stopped by adding 2 mL heptane and 1 mL saline. After centrifugation, 1.0 mL of the upper heptane phase was removed, dried under nitrogen and the acylglycerol fractions were resolved by thin-layer chromatography.

### 2.4. Determination of TG and DG Contents of WAT

Chloroform/methanol extracts from 30 mg of perigonadal WAT were resolved by thin-layer chromatography. TG and DG contents were measured as described [35].

### 2.5. Purification of HSL Protein

Recombinant human adipose HSL was produced using the Bac-to-Bac Baculovirus Expression System (version C, Invitrogen, Carlsbad, CA, USA) according to the manufacturer’s protocol. Briefly, a full-length human adipose *Lipe* cDNA flanked by *Bam*HI and *Eco*RI cloning sites [36] was cloned into *Bam*HI and *Eco*RI-digested pFastBacTMHTA so as to place the human adipose *Lipe* cDNA in frame with the upstream 6xHis tag. A recombinant bacmid was produced in DH10Bac *Escherichia coli* cells and used to transfect Sf9 insect cells. The human adipose HSL, including an N-terminal His tag, was purified using Ni sepharoseTM 6 fast Flow (GE Healthcare, Baie d’Urfe Quebec, Canada) from the transfected Sf9 cells as described [36]. Finally, the His tag was removed by Tobacco Etch Virus (TEV) protease digestion as described [36].

### 2.6. Western Blot

Western blots were performed as described [35] with anti-β-actin(#3700), anti-ATGL(#2138), anti-phospho-Ser563 HSL(#4139) and anti-phospho-Ser660 HSL(#4126) from Cell Signaling (Danvers, MA, USA), and anti-GRP78 (ab21685) and anti-DGAT2 (ab237613) from abcam (Shanghai, China). The polyclonal antibody to mouse HSL was prepared as described [37]. Levels of individual proteins were estimated from Western blots using Image J software (ImageJ 1.52a, Wayne Rasband, National Institutes of Health, Bethesda, MD, USA).

### 2.7. Statistical Analysis

Values are presented as means ± SEM. Statistical comparisons for two groups were performed by the Student *t* test and for more than two groups, by ordinary 1-way ANOVA followed by Tukey’s multiple comparison testing with GraphPad Prism 6 software (GraphPad Software, Inc., La Jolla, CA, USA), * *p* < 0.05; ** *p* < 0.01; *** *p* < 0.001.

## 3. Results

### 3.1. Adipose HSL Has TG Hydrolase Activity both in the Presence and Absence of ATGL

**Figure 1:**
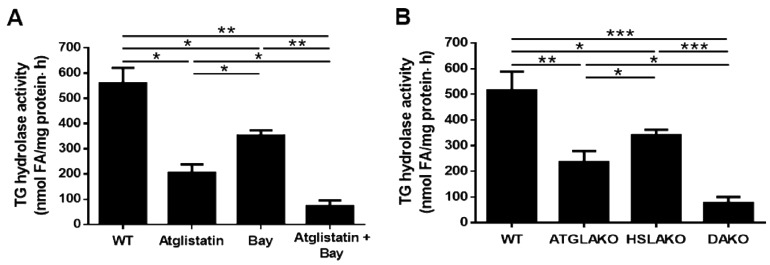
TG hydrolase activities of white adipose tissue (WAT). Triglyceride (TG) hydrolase activities were determined in adipose tissue from (**A**) 24-h fasted 10-week-old male WT mice in the presence or absence of the specific inhibitor of adipose triglyceride lipase (ATGL), Atglistatin (40 μM) and/or the specific inhibitor of HSL, Bay (50 nM). (n = 6) (**B**) from 24-h fasted 10-week-old male WT mice, adipose ATGL-deficient (ATGLAKO) mice, adipose HSL-deficient (HSLAKO) mice, and adipose ATGL and HSL double deficient (DAKO) mice. Tri[9,10 (n)-3H]triolein was used as the substrate (n = 6 mice). Values are mean ± S.E.M. * *p* < 0.05, ** *p* < 0.01, *** *p* < 0.001.

Purified HSL can hydrolyze TGs *in vitro*, although at a lesser rate than that of DG hydrolysis [38]. To test whether adipose HSL could hydrolyze TGs in the absence of ATGL, TG hydrolase activity was measured using radiolabeled triolein as a substrate in the presence of specific inhibitors of either ATGL or HSL alone or in combination. Compared to control adipose tissue, TG hydrolase activity was low in the presence of HSL inhibition, lower with ATGL inhibition, and lowest when both ATGL and HSL were inhibited (Figure 1A). This was also measured in the adipose tissue from adipose ATGL-deficient (ATGLAKO) mice, adipose HSL-deficient (HSLAKO) mice and the double adipose ATGL- and HSL-deficient (DAKO) mice (Figure 1B). These results suggest that adipose HSL has TG hydrolase activity both in the absence and presence of ATGL.

### 3.2. Adipose ATGL Functions as a Transacylase in the Absence of HSL in 24-h Fasted Mice

**Figure 2:**
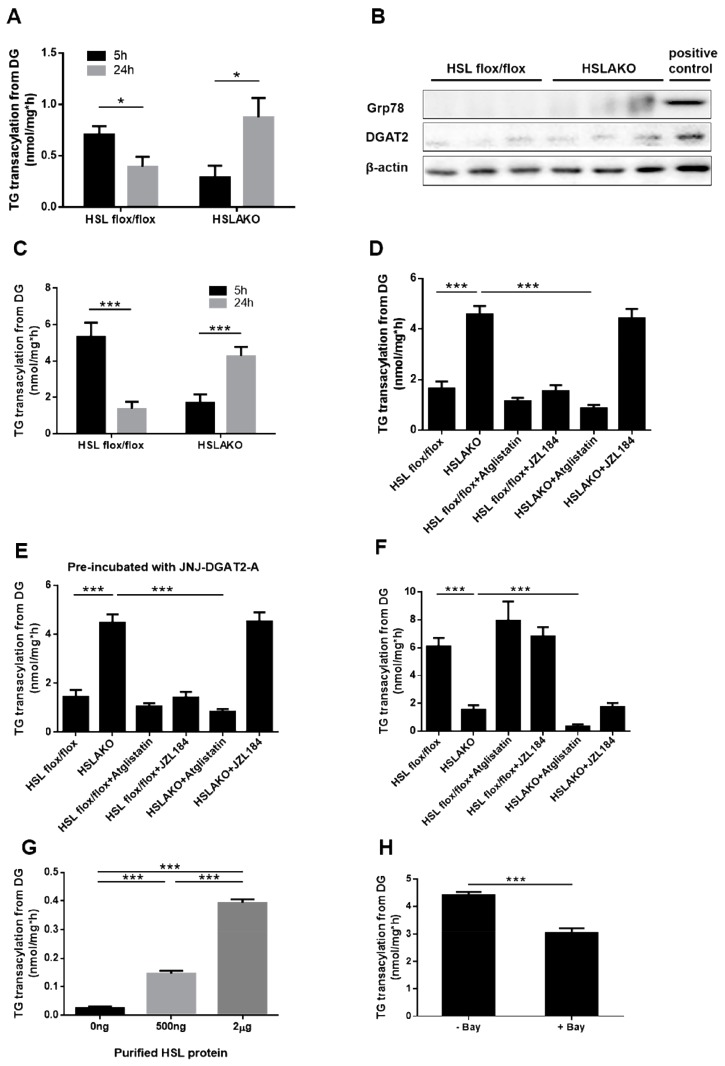
Levels of ^3^H-TG transacylation from ^3^H-DGs. (**A**) Levels of ^3^H-TG transacylation from ^3^H-DGs in WAT cytosol from 5-h and 24-h fasted mice. (**B**) Levels of DGAT2 and ER marker Grp78 protein in the isolated lipid droplet (LD) fraction of HSL flox/flox and HSLAKO mice were measured by Western blotting. Positive control means cytosolic protein from HSL flox/flox mice. (**C**) Levels of ^3^H-TG transacylation from ^3^H-DGs in WAT lipid droplet extracts from 5-h and 24-h fasted mice. (**D**) Levels of ^3^H-TG transacylation from ^3^H-DGs in WAT lipid droplet extracts from 24-h fasted mice in the presence or absence of ATGL inhibitor and MGL inhibitor. (**E**) Levels of ^3^H-TG transacylation from ^3^H-DGs in WAT lipid droplet extracts, which were pre-incubated with DGAT2 inhibitor, from 24-h fasted mice in the presence or absence of ATGL inhibitor and MGL inhibitor. (**F**) Levels of ^3^H-TG transacylation from ^3^H-DGs in WAT lipid droplet extracts from 5-h fasted mice in the presence or absence of ATGL inhibitor and MGL inhibitor. (**G**) Levels of ^3^H-TG transacylation from ^3^H-DGs in the presence of the indicated amounts of purified HSL protein. (**H**) Levels of ^3^H-TG transacylation from ^3^H-DGs in WAT lipid droplet extracts in the presence or absence of the HSL inhibitor, Bay. HSLAKO: adipose specific HSL deficient mice. Atglistatin (40 μM), ATGL inhibitor; Bay (50 nM), HSL inhibitor; JZL184 (1 μM), MGL inhibitor; JNJ-DGAT2-A (50 µM), DGAT2 inhibitor. (n = 6 mice under each condition.). Values are mean ± S.E.M. * *p* < 0.05, ** *p* < 0.01, *** *p* < 0.001.

HSL is the only known DG hydrolase in adipose tissue. In the absence of HSL, can ATGL act upon DG? ATGL protein has very low DG hydrolase activity in vitro [15]. However, ATGL reportedly has transacylation activity [29], by which fatty acyl groups could transfer between acylglycerol molecules without FA release. In the absence of HSL, high levels of DG accumulate in adipose tissue [2]. We hypothesized that ATGL may act as a transacylase under these conditions, transferring an acyl group between two DG molecules to form one TG and one MG. If this occurs, TG production from DGs is predicted to be high in HSL-deficient WAT. To test this hypothesis directly, we first performed an in vitro incubation of WAT-derived cytosolic proteins with radiolabeled DGs. The production of TG was significantly higher in samples from 24-h fasted HSLAKO mice than in control samples (*p* < 0.01) (Figure 2A). TG synthesis is usually catalyzed by acyl CoA:diacylglycerol acyltransferase (DGAT) enzymes, DGAT1 or DGAT2 [39,40]. To explore whether DG re-esterification in HSLAKO cytosolic protein was mainly dependent on DGATs, which are highly expressed in ER, WAT-derived lipid droplet protein was extracted. The removal of ER was confirmed by the Western blot of Grp78, a marker of ER (Figure 2B). Protein levels of DGAT2 were also tested and showed a weak signal (Figure 2B). The extracted lipid droplet protein was incubated with radiolabeled DG. The result showed TG transacylation from DG is significantly higher in 24-h fasted HSLAKO extracts than in controls (*p* < 0.01) (Figure 2C) and it was even higher than that of cytosol. To clarify whether the residual DGAT2 in the lipid droplet fraction contributes to TG synthesis, we subsequently performed TG transacylation from DG in the presence of the DGAT2 inhibitor (JNJ-DGAT2-A, 50 µM). Similar patterns of TG transacylation from DG were observed with and without the inhibitor (Figure 2D,E), suggesting that DG to TG formation in HSLAKO mice is DGAT-independent.

After exclusion of the involvement of DGAT2, we next tried to determine whether ATGL acts as a transacylase in the absence of HSL. WAT lipid droplet protein was extracted and was incubated with radiolabeled DG. The results showed that in the samples from 24-h fasted mice, TG transacylation from DG is significantly higher in HSLAKO extracts than in control extracts (*p* < 0.01). Importantly, this increase was abolished in the presence of the ATGL inhibitor Atglistatin, but not by the MGL inhibitor JZL184 (Figure 2D), suggesting that TG transacylation from DGs of HSLAKO mice was ATGL-mediated. Levels of TG transacylation from DG were similar between samples from control mice with or without the incubation of ATGL or MGL inhibitors, suggesting that in the presence of HSL, neither ATGL nor MGL mediates a substantial rate of transacylation. These results are consistent with the hypothesis that ATGL acts as a transacylase in the adipose tissue of fasting HSLAKO mice.

### 3.3. Adipose HSL Shows Transacylase Activity in 5-h Fasted Mice

Because ATGL-mediated transacylation is high in 24-h fasted HSLAKO mice as shown in Figure 2A,C, and since the rate of lipolysis normally increases with fasting, we predicted that transacylation activity might be low in 5-h fasted mice. Surprisingly, TG synthesis from radiolabeled DG, measured after 5 h of fasting, was significantly lower in adipose tissue from HSLAKO mice than in that of controls (Figure 2F), the opposite finding to observations after 24 h of fasting. These results suggest that HSL may contribute to normal TG synthesis after short fasting. To further test for TG synthesis activity in HSL, purified HSL protein was incubated with radiolabeled DG. Transacylation to TG from DG was significantly higher than in non-protein controls and it increased in proportion to the amount of HSL protein (Figure 2G). This was also tested in the adipose lipid droplet fraction of wild type mice, incubated with the HSL inhibitor Bay. TG transacylation from DG was lower in the presence of Bay than in non-inhibited controls (Figure 2H). Together, these results suggest that HSL has transacylation activity that is active in adipose tissue extracts from 5-h fasted mice. In 5-h fasted mice, WAT lipid droplet protein, inhibition of ATGL or MGL in the adipose tissue of normal mice produced similar levels of TG transacylation from DG to those of controls, suggesting that when HSL is present, ATGL and MGL do not contribute to TG transacylation from DG. However, when ATGL inhibitor was added to the adipose tissue of HSLAKO mice, TG transacylation from DG decreased significantly (Figure 2F). Incubation with the MGL inhibitor JZL184 did not detectably influence TG transacylation from DG in HSLAKO mice. Together, these results suggest that after a 5-h fast, ATGL also mediates TG synthesis by transacylation in HSLAKO mice.

### 3.4. Transacylation Activity of HSL Is Phosphorylation Independent

**Figure 3:**
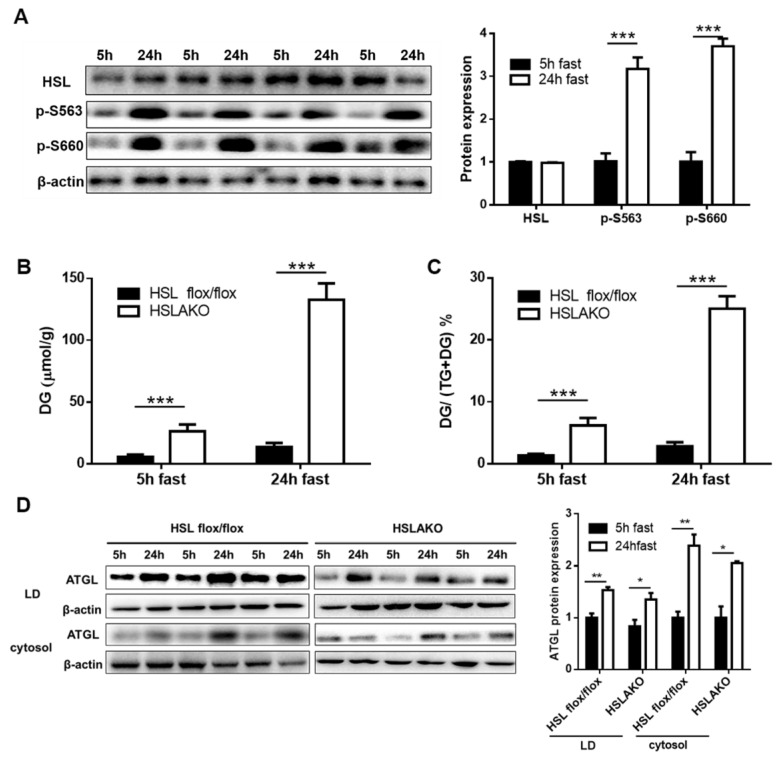
Levels of HSL protein, HSL phosphorylation, DG and of the DG/(TG + DG) ratio, and ATGL protein in WAT of 5-h and 24-h fasted mice. (**A**) Levels of HSL and HSL phosphorylation. Protein was extracted from WAT of wild type mice. Expression levels of total HSL, and of two phosphorylated forms of HSL, HSL-Ser563, and HSL-Ser660, were measured by Western blotting. The bar graphs show the quantification of blots from four different mice (mean ± SEM) by image J software. In each case, the level of HSL at 5 h of fasting is assigned a value of unity and the level at 24 h of fasting is expressed relative to it. (**B**) Measurement of DG content and (**C**) of the DG/(TG + DG) ratio of mouse WAT after 5 h or 24 h of fasting. (**D**) Levels of ATGL protein in the lipid droplet and cytosolic fractions of HSL flox/flox and HSLAKO mice after 5 h or 24 h of fasting were detected by Western blotting. Results were quantified from three different mice in each group. Values are mean ± S.E.M. * *p* < 0.05, ** *p* < 0.01, *** *p* < 0.001.

To understand why the transacylation activity of HSL was seen after a 5-h but not after a 24-h fast, we predicted that HSL may perform different functions in different nutritional states. Its DG hydrolase activity is phosphorylation dependent [41]. We therefore examined the levels of HSL protein and of HSL phosphorylation in adipose tissue. Total adipose HSL protein was similar at 5 h and at 24 h of fasting (Figure 3A). However, the phosphorylation of HSL Ser563 and Ser660 was significantly higher after 24 h than after 5 h of fasting (Figure 3A). These results suggest that non-phosphorylated HSL may mediate transacylation, and that this activity is not directly related to that of DG hydrolysis.

### 3.5. Higher Levels of ATGL-Mediated Transacylation in 24-h Fasted Than in 5-h Fasted HSL-Deficient Adipose Tissues Relate both to Substrate Availability and to ATGL Level

After a 24-h fast, ATGL-mediated transacylation in adipose tissue from HSLAKO mice is higher than in controls but after 5 h, transacylation is lower than in controls. To explore these opposite changes, we tested whether higher substrate availability and higher ATGL level may each contribute. In all cases, adipose DG levels were higher in HSLAKO mice than in controls. In HSLAKO mice, we found greater DG accumulation after 24 h than after 5 h of fasting: 130.7 ± 13.2 versus 26.5 ± 6.2 µmol/g, a 4.9-fold difference (*p* < 0.001, Figure 3B). DG fractions, i.e., DG/ (TG + DG), in 24-h and 5-h fasted HSLAKO WAT were 25.56 ± 1.99% and 6.21 ± 1.57% (*p* < 0.001), versus 2.84 ± 0.61% and 1.34 ± 0.25% in control WAT (*p* > 0.05, Figure 3C). Moreover, levels of ATGL protein in LD and cytosolic fractions after 5 h and 24 h of fasting were detected by Western blot. As expected, ATGL is more abundant in LD than in the cytosolic fraction (Figure 3D), a possible explanation for higher TG transacylation from DG in LD than in whole homogenate as shown in Figure 2A,C. Also, higher levels of ATGL were shown in the LD fraction of 24 h of fasting than that of 5 h of fasting (Figure 3D), a possible explanation for increased TG transacylation from DG in 24 h of fasting than in 5 h of fasting HSLAKO mice as shown in Figure 2D,F. These data suggest that ATGL-mediated transacylation relates both to the availability of its DG substrate and to the level of ATGL protein.

## 4. Discussion

**Figure 4:**
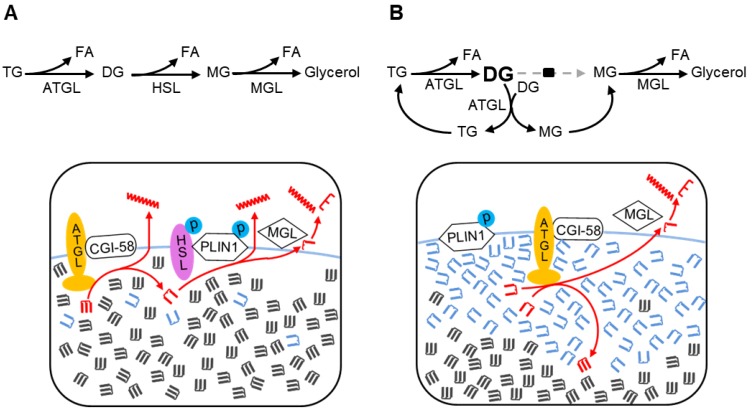
Two pathways of lipolysis. (**A**). The canonical pathway of adipocyte lipolysis. The canonical lipolytic pathway is shown on top and is diagrammed in red below. The first step is TG hydrolysis by ATGL. Next, active phosphorylated HSL on the lipid droplet surface cleaves DGs, maintaining a low level of DGs at the lipid droplet surface. ATGL has transacylation capacity but, under conditions of low DG concentrations, this accounts for a negligible fraction of acylglycerol flux. Monoglycerols are cleaved by MGL. (**B**) The transacylation pathway of adipocyte lipolysis in fasting HSL-deficient WAT. In this pathway, ATGL catalyzes both TG hydrolysis and transacylation of DGs. ATGL-mediated TG hydrolysis in HSL-deficient adipocytes results in high levels of DGs. DGs are less hydrophobic than TGs and hence concentrate at the lipid droplet surface, near ATGL. From two DG molecules, transacylation produces one MG (a substrate for MGL) and one TG that can be cleaved by the lipase activity of ATGL. In HSL deficiency, the high local concentration of DGs is predicted to enhance transacylation by ATGL, which mediates a major fraction of TG turnover and acylglycerol flux under these conditions. DGs are shown in blue; TGs, in dark grey.

We recently showed that an epistatic interaction exists between the *Pnpla2* and *Lipe* genes that encode ATGL and HSL, respectively [3]. However, the mechanism of this interaction is unknown. In this study, we showed that the catalytic activities of ATGL and HSL overlap with each other physiologically: adipose HSL can provide TG hydrolase activity in the absence of ATGL and adipose ATGL can metabolize DGs by transacylation when HSL is deficient. This functional redundancy provides alternative lipolytic pathways for energy metabolism when either ATGL or HSL is absent, but not if both are deficient. Also, for the first time, we show that HSL has transacylase activity, which could contribute to normal TG synthesis in adipose tissue. The discovery of these previously unsuspected pathways of acylglycerol homeostasis in adipose tissues shows that the view of adipose lipolysis as a sequential linear pathway, although useful, is a simplification of the physiological situation (Figure 4A).

Previous studies have demonstrated transacylation activity in mammalian tissue (intestine) [27,28], and have shown transacylation activity in vitro with purified ATGL [29]. However, it has been unclear whether ATGL can perform transacylation in vivo. In the present study, fasting HSL-deficient WAT provides optimal conditions for the detection of transacylation. At the lipid droplet surface of HSL-deficient WAT, ATGL is confronted with a high concentration of DG and a correspondingly lower level of TGs (Figure 4B). We demonstrate a high level of in vitro TG transacylation from DGs in HSL-deficient WAT. Furthermore, this is not observed in the presence of ATGL inhibition, suggesting that ATGL mediates fasting-related transacylation. Interestingly, ATGL-mediated transacylation in HSLAKO adipose tissue is greater after a 24-h than after a 5-h fast, relating both to an increase in ATGL enzyme level and to an increase in the concentration of DGs. This pattern is similar to the normal physiological increase in lipolysis with fasting [7] Together, these results show that in the absence of HSL, ATGL functions as a transacylase in adipose tissue. The consequence of ATGL-mediated transacylation is the creation of a cyclical, HSL-independent alternative lipolytic pathway.

We considered whether TG might arise in other enzyme-mediated or non-enzymatic chemical reactions. Most TG synthesis in adipose tissue is mediated by DGAT enzymes [40,42], which require acyl-CoA and ATP as substrates and which are bound to the membrane of the endoplasmic reticulum. Under the experimental conditions described in this article, endogenous sources of the soluble substrates of DGAT were removed during gradient centrifugation and ER fragments were also eliminated by the subcellular fractionation used to enrich for lipid droplets. Therefore, the observed TG transacylation from DGs is not attributable to a contamination with DGAT. Nonenzymatic transacylation is a theoretical consideration, but DG to TG synthesis was undetectable under protein-free conditions, suggesting that nonenzymatic transacylation does not occur to a measurable extent under the conditions tested.

Physiologically, ATGL-mediated transacylation is a potential mechanism to explain the occurrence of lipolysis in HSL-deficient patients [43] and mice [44,45]. Although the full clinical spectrum of human HSL deficiency remains to be defined, there are many similarities between reported findings in homozygous HSL-deficient patients and HSL-deficient mice. Both progressively develop partial lipodystrophy [43,45] and both have elevated levels of DG in adipose tissue. Symptoms of deficient lipolysis, such as fasting intolerance, have not been mentioned to date in HSL-deficient humans. We speculate that transacylation-mediated lipolysis by ATGL may protect HSL-deficient patients from fasting energy deficiency. The data presented here may help to guide future clinical studies of lipolysis in HSL-deficient humans that might explore the role, if any, of transacylation in this process.

ATGL-mediated transacylation may also be pertinent in other organs and other species. For instance, perhaps in brown adipose tissue (BAT). In most cases, lipolysis and TG synthesis were extensively studied in WAT due to its systemic importance [21,46,47,48,49,50]. Even the fatty acids used for thermogenesis in BAT are primarily derived from WAT lipolysis [51,52]. In DAKO mice, liposarcoma occurs in brown adipose tissue (BAT) with 100% incidence. Although both ATGL and HSL are highly expressed in WAT and BAT, whether they perform similar functions in BAT as in WAT needs further investigation. Birds reportedly do not express HSL [21,53,54], but have a high metabolic rate and rely heavily on fat energy stores during seasonal migration. Hepatic lipolysis is a potential factor in the current epidemic of hepatic steatosis [55]. We have shown that HSL does not contribute substantially to DG or TG hydrolase activities in mouse liver [2]. How does lipolysis occur in liver independently of HSL? Possibilities include the presence of an undiscovered lipase or ATGL-mediated transacylation and lipolysis. We observed that ATGL-deficient mouse livers accumulate not only TG but also DG [4], suggesting that the transacylation may be favored in liver as in HSL-deficient adipose tissue.

HSL provides the only substantial DG hydrolase activity in adipose tissue [19]. The DG hydrolase activity of HSL is phosphorylation dependent [41]. However, HSL shows a low level of phosphorylation in conditions under which transacylation is most active in adipose tissue. Therefore, the DG hydrolase and transacylation activities of HSL are controlled differently. Further investigations are needed to determine the contribution of HSL-mediated transacylation to adipose tissue TG synthesis in vivo.

In conclusion, this work reveals a previously unrecognized transacylation-mediated cyclic pathway of lipolysis in adipose tissue. Transacylation allows for rerouting and the maintenance of lipolytic flux in HSL deficiency and provides an example of physiological resilience despite the complete deficiency of a key enzyme of energy metabolism.

## References

[1] Zechner R., Madeo F., Kratky D. (2017). Cytosolic lipolysis and lipophagy: Two sides of the same coin. Nat. Rev. Mol. Cell Biol..

[2] Xia B., Cai G.H., Yang H., Wang S.P., Mitchell G.A., Wu J.W. (2017). Adipose tissue deficiency of hormone-sensitive lipase causes fatty liver in mice. PLoS Genet..

[3] Wu J.W., Preuss C., Wang S.P., Yang H., Ji B., Carter G.W., Gladdy R., Andelfinger G., Mitchell G.A. (2017). Epistatic interaction between the lipase-encoding genes pnpla2 and lipe causes liposarcoma in mice. PLoS Genet..

[4] Wu J.W., Wang S.P., Alvarez F., Casavant S., Gauthier N., Abed L., Soni K.G., Yang G., Mitchell G.A. (2011). Deficiency of liver adipose triglyceride lipase in mice causes progressive hepatic steatosis. Hepatology.

[5] Halldin M.U., Forslund A., von Dobeln U., Eklund C., Gustafsson J. (2007). Increased lipolysis in lchad deficiency. J. Inherit. Metab Dis..

[6] Spiekerkoetter U., Lindner M., Santer R., Grotzke M., Baumgartner M.R., Boehles H., Das A., Haase C., Hennermann J.B., Karall D. (2009). Treatment recommendations in long-chain fatty acid oxidation defects: Consensus from a workshop. J. Inherit. Metab. Dis..

[7] Wu J.W., Yang H., Wang S.P., Soni K.G., Brunel-Guitton C., Mitchell G.A. (2015). Inborn errors of cytoplasmic triglyceride metabolism. J. Inherit. Metab. Dis..

[8] Leen W.G., Taher M., Verbeek M.M., Kamsteeg E.J., van de Warrenburg B.P., Willemsen M.A. (2014). Glut1 deficiency syndrome into adulthood: A follow-up study. J. Neurol..

[9] Daci A., Bozalija A., Jashari F., Krasniqi S. (2018). Individualizing treatment approaches for epileptic patients with glucose transporter type1 (glut-1) deficiency. Int. J. Mol. Sci..

[10] Crenn P., Maillot F. (2007). [dietary advice for treatment of inborn errors of metabolism in adult neurology: Principes and limitations]. Rev. Neurol..

[11] Stenlid M.H., Ahlsson F., Forslund A., von Dobeln U., Gustafsson J. (2014). Energy substrate metabolism in pyruvate dehydrogenase complex deficiency. J. Pediatr. Endocrinol. Metab..

[12] Attane C., Peyot M.L., Lussier R., Poursharifi P., Zhao S., Zhang D., Morin J., Pineda M., Wang S., Dumortier O. (2016). A beta cell atgl-lipolysis/adipose tissue axis controls energy homeostasis and body weight via insulin secretion in mice. Diabetologia.

[13] Schreiber R., Diwoky C., Schoiswohl G., Feiler U., Wongsiriroj N., Abdellatif M., Kolb D., Hoeks J., Kershaw E.E., Sedej S. (2017). Cold-induced thermogenesis depends on atgl-mediated lipolysis in cardiac muscle, but not brown adipose tissue. Cell Metab..

[14] Martinez-Lopez N., Garcia-Macia M., Sahu S., Athonvarangkul D., Liebling E., Merlo P., Cecconi F., Schwartz G.J., Singh R. (2016). Autophagy in the cns and periphery coordinate lipophagy and lipolysis in the brown adipose tissue and liver. Cell Metab..

[15] Zimmermann R., Strauss J.G., Haemmerle G., Schoiswohl G., Birner-Gruenberger R., Riederer M., Lass A., Neuberger G., Eisenhaber F., Hermetter A. (2004). Fat mobilization in adipose tissue is promoted by adipose triglyceride lipase. Science.

[16] Belfrage P., Jergil B., Stralfors P., Tornqvist H. (1977). Hormone-sensitive lipase of rat adipose tissue: Identification and some properties of the enzyme protein. FEBS Lett..

[17] Tornqvist H., Belfrage P. (1976). Purification and some properties of a monoacylglycerol-hydrolyzing enzyme of rat adipose tissue. J. Biol. Chem..

[18] Haemmerle G., Lass A., Zimmermann R., Gorkiewicz G., Meyer C., Rozman J., Heldmaier G., Maier R., Theussl C., Eder S. (2006). Defective lipolysis and altered energy metabolism in mice lacking adipose triglyceride lipase. Science.

[19] Haemmerle G., Zimmermann R., Hayn M., Theussl C., Waeg G., Wagner E., Sattler W., Magin T.M., Wagner E.F., Zechner R. (2002). Hormone-sensitive lipase deficiency in mice causes diglyceride accumulation in adipose tissue, muscle, and testis. J. Biol. Chem..

[20] Taschler U., Radner F.P., Heier C., Schreiber R., Schweiger M., Schoiswohl G., Preiss-Landl K., Jaeger D., Reiter B., Koefeler H.C. (2011). Monoglyceride lipase deficiency in mice impairs lipolysis and attenuates diet-induced insulin resistance. J. Biol. Chem..

[21] Zechner R., Zimmermann R., Eichmann T.O., Kohlwein S.D., Haemmerle G., Lass A., Madeo F. (2012). Fat signals--lipases and lipolysis in lipid metabolism and signaling. Cell Metab..

[22] Wu J.W., Wang S.P., Casavant S., Moreau A., Yang G.S., Mitchell G.A. (2012). Fasting energy homeostasis in mice with adipose deficiency of desnutrin/adipose triglyceride lipase. Endocrinology.

[23] Holm C., Osterlund T. (1999). Hormone-sensitive lipase and neutral cholesteryl ester lipase. Methods Mol. Biol..

[24] Mayer N., Schweiger M., Romauch M., Grabner G.F., Eichmann T.O., Fuchs E., Ivkovic J., Heier C., Mrak I., Lass A. (2013). Development of small-molecule inhibitors targeting adipose triglyceride lipase. Nat. Chem. Biol..

[25] Claus T.H., Lowe D.B., Liang Y., Salhanick A.I., Lubeski C.K., Yang L., Lemoine L., Zhu J., Clairmont K.B. (2005). Specific inhibition of hormone-sensitive lipase improves lipid profile while reducing plasma glucose. J. Pharmacol. Exp. Ther..

[26] Lowe D.B., Magnuson S., Qi N., Campbell A.M., Cook J., Hong Z., Wang M., Rodriguez M., Achebe F., Kluender H. (2004). In vitro sar of (5-(2h)-isoxazolonyl) ureas, potent inhibitors of hormone-sensitive lipase. Bioorg. Med. Chem. Lett..

[27] Lehner R., Kuksis A. (1993). Triacylglycerol synthesis by an sn-1,2(2,3)-diacylglycerol transacylase from rat intestinal microsomes. J. Biol. Chem..

[28] Buhman K.K., Smith S.J., Stone S.J., Repa J.J., Wong J.S., Knapp F.F., Burri B.J., Hamilton R.L., Abumrad N.A., Farese R.V. (2002). Dgat1 is not essential for intestinal triacylglycerol absorption or chylomicron synthesis. J. Biol. Chem..

[29] Jenkins C.M., Mancuso D.J., Yan W., Sims H.F., Gibson B., Gross R.W. (2004). Identification, cloning, expression, and purification of three novel human calcium-independent phospholipase a2 family members possessing triacylglycerol lipase and acylglycerol transacylase activities. J. Biol. Chem..

[30] Ding Y., Zhang S., Yang L., Na H., Zhang P., Zhang H., Wang Y., Chen Y., Yu J., Huo C. (2013). Isolating lipid droplets from multiple species. Nat. Protoc..

[31] Soni K.G., Lehner R., Metalnikov P., O’Donnell P., Semache M., Gao W., Ashman K., Pshezhetsky A.V., Mitchell G.A. (2004). Carboxylesterase 3 (ec 3.1.1.1) is a major adipocyte lipase. J. Biol. Chem..

[32] Irshad Z., Dimitri F., Christian M., Zammit V.A. (2017). Diacylglycerol acyltransferase 2 links glucose utilization to fatty acid oxidation in the brown adipocytes. J. Lipid Res..

[33] Zhang X., Thayer S.A. (2018). Monoacylglycerol lipase inhibitor jzl184 prevents hiv-1 gp120-induced synapse loss by altering endocannabinoid signaling. Neuropharmacology.

[34] Pan B., Wang W., Long J.Z., Sun D., Hillard C.J., Cravatt B.F., Liu Q.S. (2009). Blockade of 2-arachidonoylglycerol hydrolysis by selective monoacylglycerol lipase inhibitor 4-nitrophenyl 4-(dibenzo[d][1,3]dioxol-5-yl(hydroxy)methyl)piperidine-1-carboxylate (jzl184) enhances retrograde endocannabinoid signaling. J. Pharmacol. Exp. Ther..

[35] Fortier M., Soni K., Laurin N., Wang S.P., Mauriege P., Jirik F.R., Mitchell G.A. (2005). Human hormone-sensitive lipase (hsl): Expression in white fat corrects the white adipose phenotype of hsl-deficient mice. J. Lipid Res..

[36] Wang S.P., Wu J.W., Bourdages H., Lefebvre J.F., Casavant S., Leavitt B.R., Labuda D., Trasler J., Smith C.E., Hermo L. (2014). The catalytic function of hormone-sensitive lipase is essential for fertility in male mice. Endocrinology.

[37] Wang S.P., Chung S., Soni K., Bourdages H., Hermo L., Trasler J., Mitchell G.A. (2004). Expression of human hormone-sensitive lipase (hsl) in postmeiotic germ cells confers normal fertility to hsl-deficient mice. Endocrinology.

[38] Rodriguez J.A., Ben Ali Y., Abdelkafi S., Mendoza L.D., Leclaire J., Fotiadu F., Buono G., Carriere F., Abousalham A. (2010). In vitro stereoselective hydrolysis of diacylglycerols by hormone-sensitive lipase. Biochim. Biophys. Acta.

[39] Liu Q., Siloto R.M., Lehner R., Stone S.J., Weselake R.J. (2012). Acyl-coa:Diacylglycerol acyltransferase: Molecular biology, biochemistry and biotechnology. Prog. Lipid Res..

[40] Yen C.L., Stone S.J., Koliwad S., Harris C., Farese R.V. (2008). Thematic review series: Glycerolipids. Dgat enzymes and triacylglycerol biosynthesis. J. Lipid Res..

[41] Anthonsen M.W., Ronnstrand L., Wernstedt C., Degerman E., Holm C. (1998). Identification of novel phosphorylation sites in hormone-sensitive lipase that are phosphorylated in response to isoproterenol and govern activation properties in vitro. J. Biol. Chem..

[42] Bell R.M., Coleman R.A. (1980). Enzymes of glycerolipid synthesis in eukaryotes. Annu. Rev. Biochem..

[43] Albert J.S., Yerges-Armstrong L.M., Horenstein R.B., Pollin T.I., Sreenivasan U.T., Chai S., Blaner W.S., Snitker S., O’Connell J.R., Gong D.W. (2014). Null mutation in hormone-sensitive lipase gene and risk of type 2 diabetes. N. Engl. J. Med..

[44] Osuga J., Ishibashi S., Oka T., Yagyu H., Tozawa R., Fujimoto A., Shionoiri F., Yahagi N., Kraemer F.B., Tsutsumi O. (2000). Targeted disruption of hormone-sensitive lipase results in male sterility and adipocyte hypertrophy, but not in obesity. Proc. Natl. Acad. Sci. USA.

[45] Wang S.P., Laurin N., Himms-Hagen J., Rudnicki M.A., Levy E., Robert M.F., Pan L., Oligny L., Mitchell G.A. (2001). The adipose tissue phenotype of hormone-sensitive lipase deficiency in mice. Obes. Res..

[46] Das S.K., Eder S., Schauer S., Diwoky C., Temmel H., Guertl B., Gorkiewicz G., Tamilarasan K.P., Kumari P., Trauner M. (2011). Adipose triglyceride lipase contributes to cancer-associated cachexia. Science.

[47] Rohm M., Sommerfeld A., Strzoda D., Jones A., Sijmonsma T.P., Rudofsky G., Wolfrum C., Sticht C., Gretz N., Zeyda M. (2013). Transcriptional cofactor tblr1 controls lipid mobilization in white adipose tissue. Cell Metab..

[48] Heine M., Fischer A.W., Schlein C., Jung C., Straub L.G., Gottschling K., Mangels N., Yuan Y., Nilsson S.K., Liebscher G. (2018). Lipolysis triggers a systemic insulin response essential for efficient energy replenishment of activated brown adipose tissue in mice. Cell Metab..

[49] Gao H., Mejhert N., Fretz J.A., Arner E., Lorente-Cebrian S., Ehrlund A., Dahlman-Wright K., Gong X., Stromblad S., Douagi I. (2014). Early b cell factor 1 regulates adipocyte morphology and lipolysis in white adipose tissue. Cell Metab..

[50] Koliwad S.K., Streeper R.S., Monetti M., Cornelissen I., Chan L., Terayama K., Naylor S., Rao M., Hubbard B., Farese R.V. (2010). Dgat1-dependent triacylglycerol storage by macrophages protects mice from diet-induced insulin resistance and inflammation. J. Clin. Investig..

[51] Shin H., Ma Y., Chanturiya T., Cao Q., Wang Y., Kadegowda A.K.G., Jackson R., Rumore D., Xue B., Shi H. (2017). Lipolysis in brown adipocytes is not essential for cold-induced thermogenesis in mice. Cell Metab..

[52] Petruzzelli M., Schweiger M., Schreiber R., Campos-Olivas R., Tsoli M., Allen J., Swarbrick M., Rose-John S., Rincon M., Robertson G. (2014). A switch from white to brown fat increases energy expenditure in cancer-associated cachexia. Cell Metab..

[53] Sato K., Seol H.S., Kamada T. (2010). Tissue distribution of lipase genes related to triglyceride metabolism in laying hens (gallus gallus). Comp. Biochem. Physiol. B Biochem. Mol. Biol..

[54] Saneyasu T., Shiragaki M., Kurachi K., Kamisoyama H., Honda K. (2013). Effects of short-term refeeding on the expression of genes involved in lipid metabolism in chicks (gallus gallus). Comp. Biochem. Physiol. B Biochem. Mol. Biol..

[55] Gluchowski N.L., Becuwe M., Walther T.C., Farese R.V. (2017). Lipid droplets and liver disease: From basic biology to clinical implications. Nat. Rev. Gastroenterol. Hepatol..

